# Separation of C18 Fatty Acid Esters and Fatty Acids Derived from Vegetable Oils Using Nanometer-Sized Covalent Organic Frameworks Incorporated in Polyepoxy Membranes

**DOI:** 10.1021/acsanm.3c00442

**Published:** 2023-04-10

**Authors:** Nimesh P. R. Ranasinghe Arachchige, Nathan W. Xiong, Ned B. Bowden

**Affiliations:** Department of Chemistry, University of Iowa, Iowa City, Iowa 52242, United States

**Keywords:** membranes, covalent organic framework, fatty acids, fatty acid esters, organic solvent nanofiltration, mixed matrix membranes

## Abstract

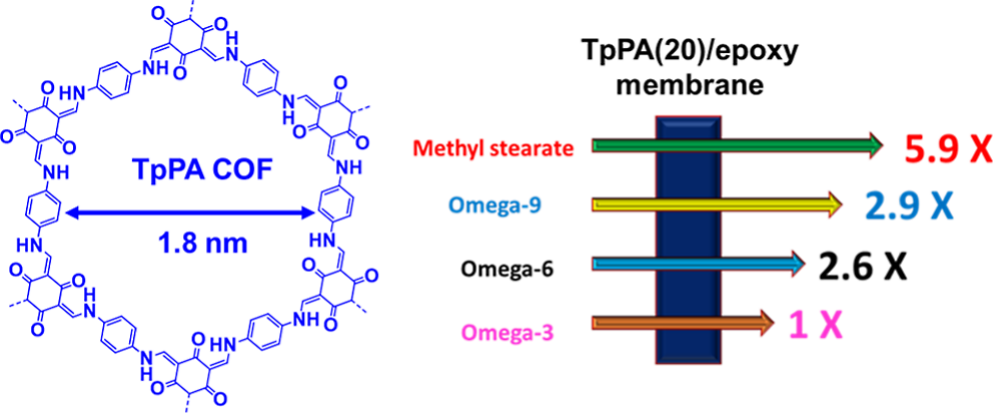

Fatty acids (FAs) and FA methyl esters (FAMEs) are easily isolated from vegetable oil and are important starting materials for the chemical industry to produce commercial products that are green, biorenewable, and nontoxic. A challenge in these applications is that mixtures of five or more FAs and FAMEs are isolated from a vegetable oil source, and methods to separate these mixtures are decades old and have increasingly high costs associated with the production of high-purity single-component FAs or FAMEs. We developed a method to separate these mixtures using mixed matrix membranes containing nanometer-sized covalent organic frameworks. The 2D, crystalline COFs possessed narrow distributions of pore sizes of 1.3, 1.8, 2.3, and 3.4 nm that separated FAs and FAMEs based on their degrees of unsaturation. The COFs were synthesized, characterized, and then encapsulated at 10 or 20% by weight into a prepolymer of epoxy that was then fully cured. For all mixed matrix membranes, as the degree of unsaturation increased, the FAs or FAMEs had a slower flux. The largest difference in flux was obtained for a COF/epoxy membrane with a pore size of 1.8 nm, and methyl stearate had a 5.9× faster flux than methyl linolenate. These are the first membranes that can separate the important C18 FAs and FAMEs found in vegetable oil.

## Introduction

**Figure 1:**
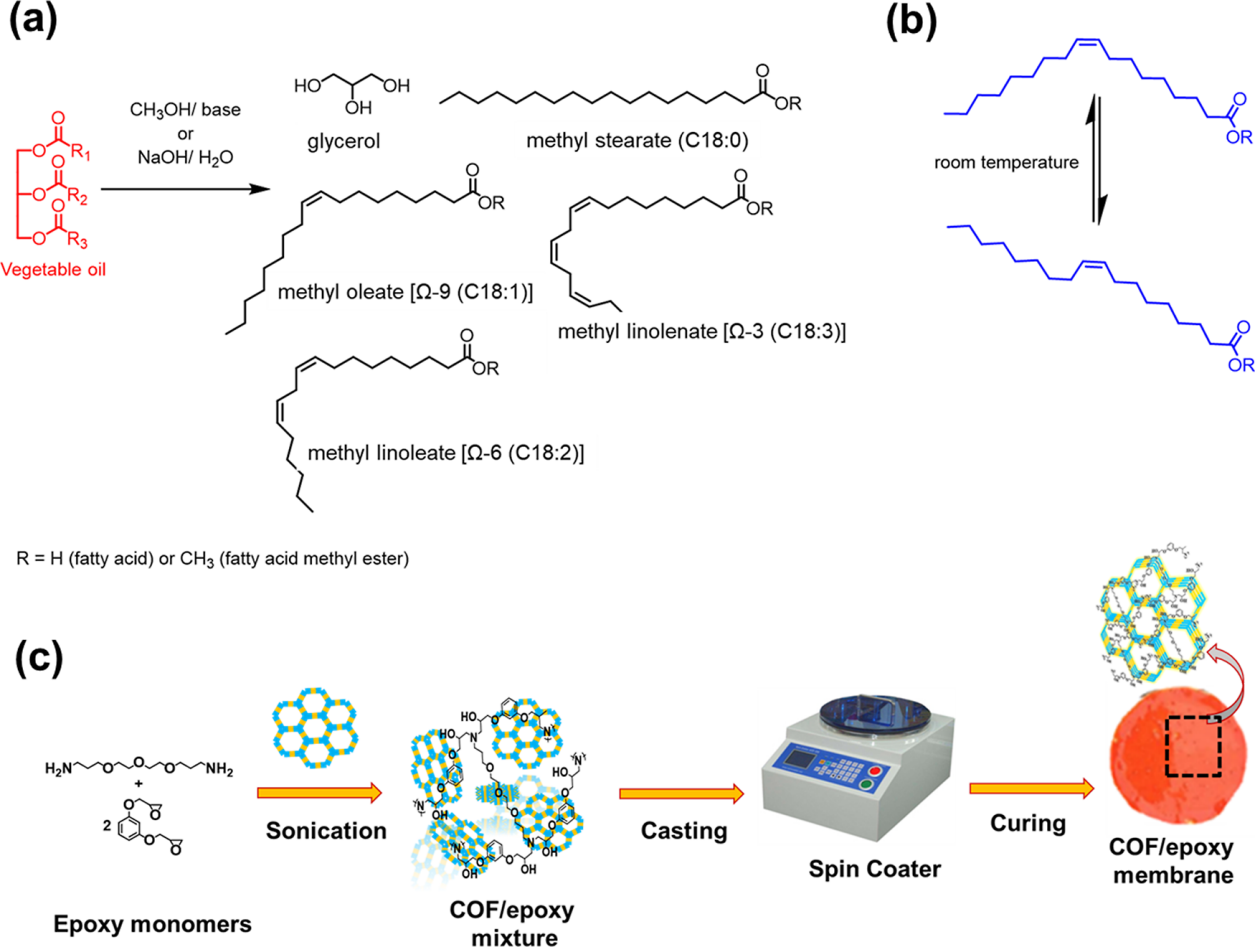
(a) Names and structures of key C18 FAMEs from vegetable oil. The C*xx*:*y* nomenclature describes the number of carbon atoms in a FA and the number of carbon–carbon double bonds. For instance, methyl linoleate [Ω-6 (C18:2)] has 18 carbon atoms in the FA and possesses 2 carbon–carbon double bonds. The omega nomenclature refers to the location of the first Csp^2^ carbon in a carbon–carbon double bond when counted from the terminal methyl. (b) Rapid rotation about carbon–carbon single bonds leads to numerous conformations that possess different energies. (c) Diagram illustrating the fabrication of COF(*n*)/epoxy membranes. The COFs were added to a prepolymer of polyepoxy, spin coated onto a solid support, and then cured to yield mixed matrix membranes.

The demand for eco-friendly products is increasing due to consumer desires for commercial products that are safe, biorenewable, nonpolluting, and recyclable.^1−3^ Most commercial products are fabricated from chemicals derived from the petroleum industry, but there is an increasing push to use natural chemicals due to the low costs of these materials and their inherent safety.^4−7^ Vegetable oils and their fatty acids (FAs) and FA methyl esters (FAMEs) are attractive feedstocks to meet these demands because they are plentiful (isolated in 200 M tons per year), inexpensive, highly reduced, and possess well-defined functional groups.^8−11^ Vegetable oils are composed of three FAs bonded together through glycerol, and most of the FAs have 16 or 18 carbons and 0, 1, 2, or 3 cis-carbon–carbon double bonds (Figure 1a).^12−14^ The number of carbon–carbon double bonds in a FA is the degree of unsaturation, and the higher the degree of unsaturation, the more prone the FA is to oxidation. The distribution of FAs in each vegetable oil is different, and the distributions for a particular vegetable oil vary slightly from growing season to growing season.^15^ The production of FAs and FAMEs is straightforward by either hydrolysis or transesterification using water or methanol under basic conditions, and FAMEs are widely used as biodiesel, animal feed, and in the production of other commercial products.^16−18^

A major challenge in the synthesis of commercial products from FAs and FAMEs is that they are isolated as mixtures of five or more different FAs and FAMEs.^13,19^ For instance, corn oil is composed of 13% saturated FAs (stearic and palmitic acids), 27% oleic acid, 58% linoleic acid, and 1% linolenic acid.^20^ These mixtures are challenging to separate to produce streams of high-purity, single-component FAs or FAMEs due to their similar physical and chemical properties, such as boiling points, solubilities, and functional groups.^11^ Methods to separate mixtures of FAs and FAMEs include distillation, chromatography, winterization, and urea clathrates, but these methods either have severe limitations in the purities that can be obtained or have high costs to yield high-purity single-component FAs or FAMEs.^21−25^ The purification of FAs is more difficult than the purification of FAMEs because they are more reactive due to the presence of carboxylic acids.^14^ The development of a low-cost method to separate mixtures of FAMEs or mixtures of FAs into streams with high-purity single components would open up new opportunities to develop commercial products.

In this paper, we report the first effective membrane separation of mixtures of C18 FAs or FAMEs using covalent organic framework (COF) mixed matrix membranes in polyepoxy (Figure 1c). Membrane separations are highly desired because they are an inexpensive method to separate chemicals and are easily scaled to industrial levels.^26−31^ Numerous examples of membranes that can separate gases and low boiling point liquids from each other have been developed and are used commercially, but there are a limited number of examples of commercial organic solvent nanofiltration (OSN) membranes.^32,33^ There have been a few reports of the separation of small, saturated FAs or FAMEs (C1–C3) utilizing a membrane process in aqueous solutions and their different solubilities.^34−36^ However, the separation of C18 FAMEs or FAs has been heavily understudied, and most reports on the separation of FAMEs have focused on their separation from triglycerides or glycerol.^37−39^ FAs and FAMEs are challenging to separate using membranes because they possess very similar sizes and shapes due to rotation about the C–C bonds (Figure 1b).^13^ In prior work, we investigated the separation of C18 FAMEs using polyepoxy membranes, but these membranes failed to separate them. The membranes also poorly separated eicosapentaenoic acid-ethyl ester (C20:5, molecular weight: 330 g mol^–1^) from docosahexaenoic acid-ethyl ester (C22:6, molecular weight: 356 g mol^–1^) with a difference in flux of only 1.4 despite a larger difference in molecular weight compared to differences between C18 FAMEs.^26^

Membranes based on COFs have emerged in the last decade as highly selective based on their crystalline structures with narrow distributions of pore sizes.^40−43^ These membranes have been used to separate gasses, ions from water, and dyes from water, but there are few applications in the field of OSN.^44−49^ We hypothesized that mixed matrix membranes of COFs suspended in a polyepoxy could separate FAs and FAMEs based on the small differences in their sizes. In this paper, we report how four different COF/polyepoxy membranes separated mixtures of FAs or FAMEs and how the pore sizes of the COFs were critically important for these separations. This is the first report of the effective separation of FAs and FAMEs using membranes and demonstrates that these important chemicals can be separated using membranes with well-defined pores.

## Experimental Section

### Materials and Methods

All chemicals were purchased from Acros, Sigma-Aldrich, Alpha Aesar, BDH, or TCI. PZ flat sheet membranes composed of poly(acrylonitrile) (molecular weight cutoff of 30,000 g mol^–1^) were purchased from Synder Filtration and used as received. ^1^H NMR spectra were collected at room temperature using a Bruker DRX-400 at 400 MHz and a Bruker DPX500 at 500 MHz. NMR samples were referenced to tetramethylsilane. Powder X-ray diffraction (PXRD) spectra were obtained using a Siemens model D5000 X-ray diffractometer (Bruker AXS Inc.) in the reflection mode using CuKα X-ray radiation (λ = 1.54 Å). The 2θ range from 2 to 35° was scanned with a scan rate of 1° min^–1^. The wide-angle XRD (WXRD) analysis of the COF(*n*)/epoxy membranes used the USAXS instrument at the Advanced Photon Source, Argonne National Laboratory.^50^ The X-ray energy was 21 keV (λ = 0.5895 Å). Scanning electron microscopy (SEM) was performed on an S-2700 SEM (Hitachi, Japan). The particles were placed on an aluminum specimen stub using adhesive carbon tape. The mount was then coated by ion sputtering with conductive gold set at 10 mA for 2.5 min and examined using SEM operated at a 2 kV accelerating voltage. Fourier transform infrared (FT-IR) spectra were collected at room temperature using an Avatar 370 FT-IR with a Diamond ATR (attenuated total reflection) in the 700–4000 cm^–1^ region.

### Synthesis of 1,3,5-Triformylphloroglucinol

A mixture of hexamethylenetetramine (15.1 g, 107.9 mmol), phloroglucinol (6.0 g, 47.6 mmol), and trifluoroacetic acid (90 mL) was placed in a dry 500 mL round-bottom flask and heated at 100 °C for 2.5 h under N_2_. Then, 150 mL of 3 M HCl was added, and the solution was heated at 100 °C for another 1 h. The reaction mixture was then allowed to cool to room temperature, the insoluble residues were removed by filtration, and the filtrate was extracted with dichloromethane (DCM) (3 × 100 mL). The organic phases were combined and dried over anhydrous Na_2_SO_4_. The desired product was obtained after removing the solvent under reduced pressure, followed by recrystallization of the crude product in ethanol, yielding a yellow powder (2.2 g, 20% yield). ^1^H NMR (500 MHz, CDCl_3_) δ = 10.15 (s, CHO), 14.13 (s, OH). ^13^C NMR (75 MHz, CDCl_3_) δ = 192.07, 173.58, and 102.87.

### Synthesis of HCOF

A Pyrex ampule (50 mL) was charged with Tp (42.0 mg, 0.2 mmol), anhydrase hydrazine (0.02 mL, 0.3 mmol), 13.6 mL of 1,4-dioxane, 1.2 mL of mesitylene, and 4.6 mL of 6 M acetic acid. The mixture was sonicated for 10 min at room temperature for homogeneous dispersion. The tube was then flash frozen at 77 K (liquid N_2_ bath) and degassed by three freeze–pump–thaw cycles. The tube was sealed off and then heated at 120 °C for 3 days. A dark red precipitate was collected by filtration and washed with methanol, dimethylformamide (DMF), and THF. The resulting powder was then immersed in anhydrous THF for 3 days. It was filtered and dried under vacuum for 24 h at room temperature to yield a dark red solid (82% isolated yield).

### Synthesis of TpPa, TpBD, and TpBDDA COFs

A pyrex ampule (25 mL) was charged with Tp (63 mg, 0.3 mmol), corresponding diamine [*p*-phenylenediamine (Pa) [48.0 mg, 0.45 mmol]; benzidine (BD) [83.0 mg, 0.45 mmol]; 4,4′-(buta-1,3-diyne-1,4-diyl) dianiline (BDDA) [105.0 mg, 0.45 mmol]], 1.5 mL of 1,4-dioxane, 1.5 mL of mesitylene, and 0.5 mL of 6 M acetic acid. The mixture was sonicated for 10 min at room temperature for homogeneous dispersion. The tube was degassed by three freeze–pump–thaw cycles. The tube was sealed off and then heated at 120 °C for 3 days for TpPA and TpBD. The vial was heated to 120 °C for 4 days for the synthesis of TpBDDA. A colored [TpPA (dark red), TpBD (yellow), and TpBDDA (orange)] precipitate was collected by filtration and washed with anhydrous acetone. The powder collected was then solvent exchanged with anhydrous acetone 5–6 times and then dried at 180 °C under a vacuum for 24 h to yield the corresponding COFs in high yields [TpPA (80%), TpBD (82%) and TpBDDA (84%)].

### Synthesis of COF Fragment (S1)

Compound S1 was synthesized according to a previously reported method. In a 100 mL round-bottom flask, Tp (100 mg, 0.48 mmol) and aniline (443 mg, 4.8 mmol) were dissolved in ethanol (30 mL). The reaction was refluxed overnight and then cooled to room temperature.^51^ The yellow precipitate was collected by filtration, washed with cold ethanol, and dried under reduced pressure to afford the product as a yellow solid (165 mg, 0.39 mmol, 80% yield). ^1^H NMR (500 MHz, CDCl_3_) δ = 13.0–13.4 (m, NH, 3H), 8.94–8.69 (m, HC-N, 3H), 7.43 (m, Ph, 6H), 7.32 (m, Ph, 6H), 7.22 (m, Ph, 3H). ^13^C NMR (75 MHz, CDCl_3_): δ 185.53, 149.31, 139.05, 129.90, 125.67, 117.68, 106.69.

### Synthesis of Epoxy Membrane

Amine (4,7,10-trioxa-1,13-tridecanediamine, 1.97 mL, 0.009 mol), epoxide (resorcinol diglycidyl ether, 4.0 g, 0.018 mol), and DMF (0.64 mL) were combined in a scintillation vial and mixed thoroughly using a Vortex-Genie2 and a Teflon stir bar. Slight vacuum was pulled on the vial to remove air bubbles created by mixing. A sample of the mixture (1.5 mL) was spread on top of a 12.5 cm × 12.5 cm square of the PZ flat sheet membrane. A small beaker of DMF (10 mL) was placed next to the membrane. A glass cover was placed over top of the membrane and the small beaker of DMF. The polymer was cured at room temperature for 72 h.

### Preparation of COF(*n*)/Epoxy Hybrid Membranes

The [COF(*n*)/epoxy] membranes, where *n* = 10 or 20 on a w/w basis, were prepared by the solution casting method. The amine (4,7,10-trioxa-1,13-tridecanediamine, 1.97 mL, 0.009 mol), epoxide (resorcinol diglycidyl ether, 4.0 g, 0.018 mol), and DMF (2.0 mL) were combined in a scintillation vial and mixed thoroughly using a Vortex-Genie2 and Teflon stir bar. A slight vacuum was pulled on the vial to remove air bubbles created by mixing. Next, the epoxy monomers in DMF were mixed with the HCOF, TpPA, TpBD, or TpBDDA (1.50 or 0.67 g) and stirred for 15 min, followed by bath sonication for 30 min to obtain homogenous suspensions. The suspensions were poured on the PZ membrane on a spin coater and spun at 2000 rpm for 3 min. A small beaker of DMF (10 mL) was placed next to the membrane. A glass cover was placed over the membrane and the beaker of DMF. The membranes were cured at room temperature for 72 h.

### Measurement of Flux of FAMEs and FAs Using ^1^H NMR Spectroscopy

In these separations, the flux of the FAMEs and FAs was quantified using ^1^H NMR spectroscopy. In each separation, methyl stearate (or stearic acid) was used with one of the unsaturated FAMEs (or FAs). This allowed the flux of both chemicals to be found using unique peaks in the NMR spectra.

### Separation of Methyl Stearate and Methyl Oleate through HCOF(*n*)/Epoxy, TpPa(*n*)/Epoxy, TpBD(*n*)/Epoxy, and TpBDDA(*n*)/Epoxy Membranes

A TpPA(20)/epoxy membrane was clamped between two glass vessels. Methyl stearate (1.07 g, 3.60 mmol), methyl oleate (1.2 mL, 3.60 mmol), and *p*-nitrobenzaldehyde (0.54 g, 3.60 mmol) were added with 30 mL of DCM to one side (retentate) of the membrane. DCM (30 mL) was added to the other side (permeate) of the membrane. On both sides of the membrane, a small amount of butylhydroxytoluene (BHT) was added as an antioxidant. The solvent on both sides of the membrane was stirred continuously at room temperature. At 12, 24, 36, 48, 60, 84, and 108 h, a 1 mL aliquot of solvent was removed from both sides of the membrane. Triphenylmethane (0.5 mL of a 0.25 M stock solution) was added to each aliquot, and the solvent was removed under vacuum. The aliquots were analyzed by ^1^H NMR spectroscopy to determine the concentrations of FAMEs and *p*-nitrobenzaldehyde. Each separation was repeated three times. The same procedure was followed for all the membranes.

### Separation of Methyl Stearate and Methyl Linoleate through HCOF(*n*)/Epoxy, TpPa(*n*)/Epoxy, TpBD(*n*)/Epoxy, and TpBDDA(*n*)/Epoxy Membranes in DCM as the Solvent

A TpPA(20)/epoxy membrane was clamped between two glass vessels. Methyl stearate (1.07 g, 3.60 mmol), methyl linoleate (1.2 mL, 3.62 mmol), and *p*-nitrobenzaldehyde (0.54 g, 3.60 mmol) were added with 30 mL of DCM to one side (retentate) of the membrane. DCM (30 mL) was added to the other side (permeate) of the membrane. On both sides of the membrane, a small amount of BHT was added as an antioxidant. The solvent on both sides of the membrane was stirred continuously at room temperature. At 12, 24, 36, 48, 60, 84, and 108 h, a 1 mL aliquot of solvent was removed from both sides of the membrane. Triphenylmethane (0.5 mL of a 0.25 M stock solution) was added to each aliquot, and the solvent was removed under vacuum. The aliquots were analyzed by ^1^H NMR spectroscopy to determine the concentrations of FAMEs and *p*-nitrobenzaldehyde. Each separation was repeated three times. The same procedure was followed for all the membranes.

### Separation of Methyl Stearate and Methyl Linolenate through HCOF(*n*)/Epoxy, TpPA(*n*)/Epoxy, TpBD(*n*)/Epoxy, and TpBDDA COFs Membranes in DCM as the Solvent

A TpPA(20)/epoxy membrane was clamped between two glass vessels. Methyl stearate (1.07 g, 3.60 mmol), methyl linolenate (1.2 mL, 3.62 mmol), and *p*-nitrobenzaldehyde (0.54 g, 3.60 mmol) were added with 30 mL of DCM to one side (retentate) of the membrane. DCM (30 mL) was added to the other side (permeate) of the membrane. On both sides of the membrane, a small amount of BHT was added as an antioxidant. The solvent on both sides of the membrane was stirred continuously at room temperature. At 12, 24, 36, 48, 60, 84, and 108 h, a 1 mL aliquot of solvent was removed from both sides of the membrane. Triphenylmethane (0.5 mL of a 0.25 M stock solution) was added to each aliquot, and the solvent was removed under vacuum. The aliquots were analyzed by ^1^H NMR spectroscopy to determine the concentrations of FAMEs and *p*-nitrobenzaldehyde. Each separation was repeated three times. The same procedure was followed for all the membranes.

### Measurement of Flux of FAs through COF/Epoxy Membranes

The flux of FAs was measured using the same method as described for the FAMEs but using a 75/25 CH_2_Cl_2_/MeOH solvent mixture.

### Flux of FAMEs and FAs through S1(20)/Epoxy Membranes

The flux of FAMEs and FAs were investigated using S1(20)/epoxy membranes using the same method as previously described for measuring the flux of FAMEs.

### Permeation of Ethyl Octanoate and Methyl Stearate through the TpPa(20)/Epoxy Membrane

A TpPa (20)/epoxy membrane was placed between two glass reservoirs, and DCM (30 mL) was added to the permeate and retentate sides of the membrane. Ethyl octanoate (0.72 mL, 3.60 mmol), methyl stearate (1.07 g, 3.60 mmol), and *p*-nitrobenzaldehyde (0.54 g, 3.60 mmol) were added to the retentate side. On both sides of the membrane, a small amount of BHT was added as an antioxidant. The solvent on both sides of the membrane was stirred continuously at room temperature. At 12, 24, 36, 48, 60, 84, and 108 h, a 1 mL aliquot of solvent was removed from the solvent on both sides of the membrane. The aliquots were used to determine the concentration and the absolute amounts of methyl stearate, ethyl octanoate, and *p*-nitrobenzaldehyde by ^1^H NMR spectroscopy after the addition of triphenylmethane (0.5 mL of a 0.25 M solution) dissolved in DCM as an internal standard.

### Permeation of Methyl (*Z*)-5-Octenoate with TpPa(20)/Epoxy Membranes

A TpPa (20)/epoxy membrane was placed between two glass reservoirs, and DCM (30 mL) was added to the permeate and retentate sides of the membrane. Methyl (*Z*)-5-octenoate (0.6 mL, 3.60 mmol), methyl stearate (1.07 g, 3.60 mmol), and *p*-nitrobenzaldehyde (0.54 g, 3.60 mmol) were added to the retentate side. On both sides, small amount of BHT was added as an antioxidant. The solvent on both sides of the membrane was stirred continuously at room temperature. At 12, 24, 36, 48, 60, 84, and 108 h, a 1 mL aliquot of solvent was removed from the solvent on both sides of the membrane. The aliquots were used to determine the concentration and the absolute amounts of the methyl stearate, methyl (*Z*)-5-octenoate, and *p*-nitrobenzaldehyde by ^1^H NMR spectroscopy after the addition of triphenylmethane (0.5 mL of a 0.25 M solution) dissolved in DCM as an internal standard.

### Purification of Methyl Linoleate from Methyl Stearate Using Multiple Extractions

A TpPa(20)/epoxy membrane was placed between two glass reservoirs, and DCM (30 mL) was added to the permeate and retentate sides of the membrane. Methyl linoleate (6.0 mL, 18.0 mmol), and methyl stearate (5.35 g, 18.0 mmol) were added to the retentate sides. On both sides of the membrane, a small amount of BHT was added as an antioxidant. The solvent on both sides of the membrane was stirred continuously at room temperature. After 48 h, the solvent in the permeate side was removed and replaced with fresh 30 mL DCM. This process was repeated four more times. After the fifth extraction, the solvent on both sides of the membrane was removed and replaced with 30 mL of fresh DCM to extract any FAMEs retained in the membrane. The amounts of methyl stearate and methyl linoleate in each extraction were determined by ^1^H NMR spectroscopy after the addition of triphenylmethane (0.5 mL of a 0.25 M solution) dissolved in DCM as an internal standard.

### Flux of Methyl Stearate and Methyl Linolenate through TpPa(20)/Epoxy Membrane Using Different Solvents

The fluxes of methyl stearate and methyl linoleate were investigated using the same procedure, as described previously, but using the solvents described in the Supporting Information.

## Results and Discussion

### Synthesis and Characterization of COFs

**Figure 2:**
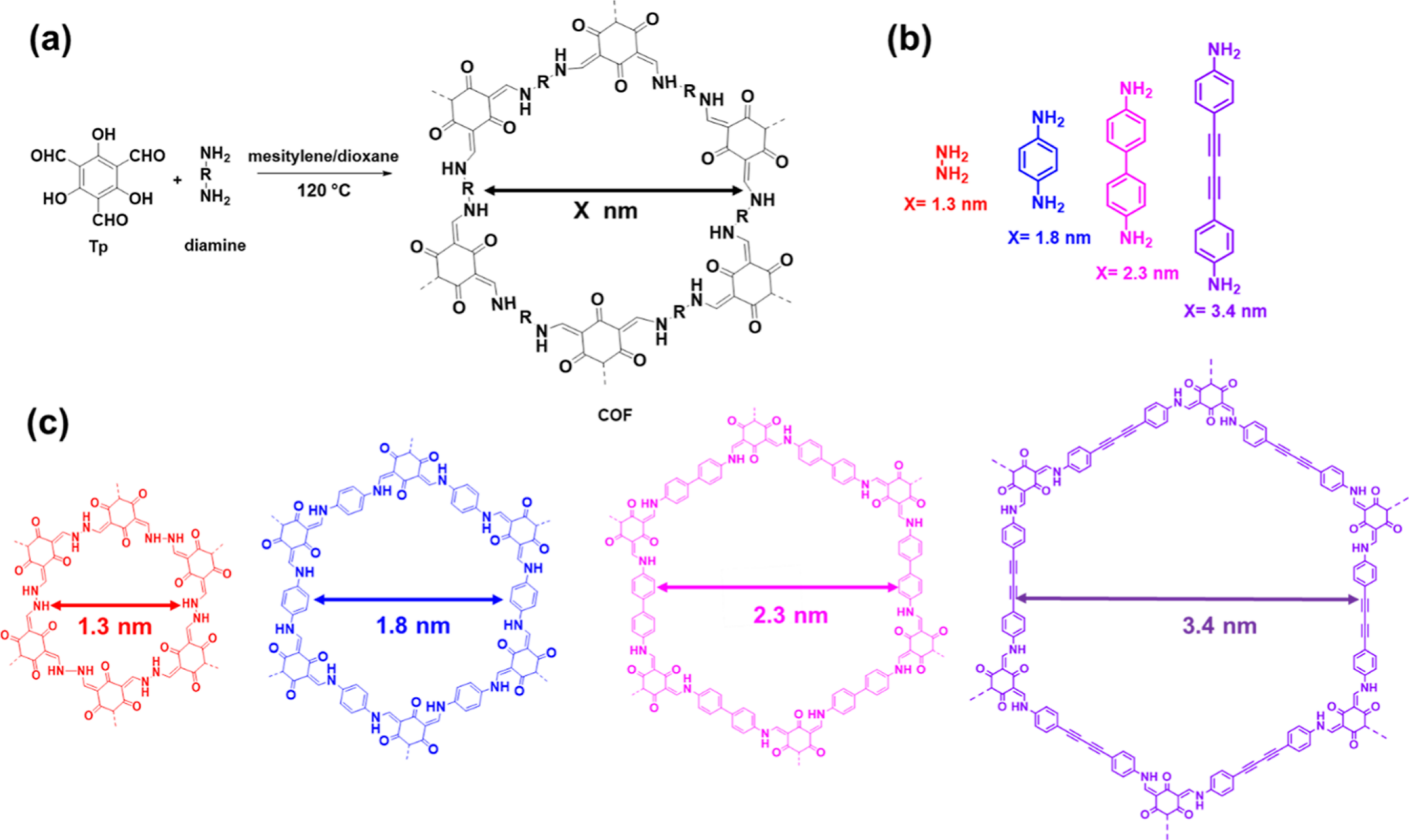
(a) COFs by the reaction of diamines with Tp to yield COFs with hexagonal pores. (b) Amines employed in the synthesis of the COFs and the corresponding size of the pores. (c) Different structures of COFs and their pore apertures.

Four COFs were synthesized by the reaction of diamines with Tp to yield imine-based COFs (Figure 2a,b). These COFs were chosen because they had dimensions that were easily varied, rapid to synthesize, and stable under a wide range of conditions, including the presence of acids, amines, alcohols, and esters.^41,45,51−53^ The COFs and their pore apertures were HCOF (1.3 nm), TpPA (1.8 nm), TpBD (2.3 nm), and TpBDDA (3.4 nm), as shown in Figure 2c. The syntheses of these COFs were reported in the literature, and slightly different syntheses were used in this work and reported here.^51,54−56^

**Figure 3:**
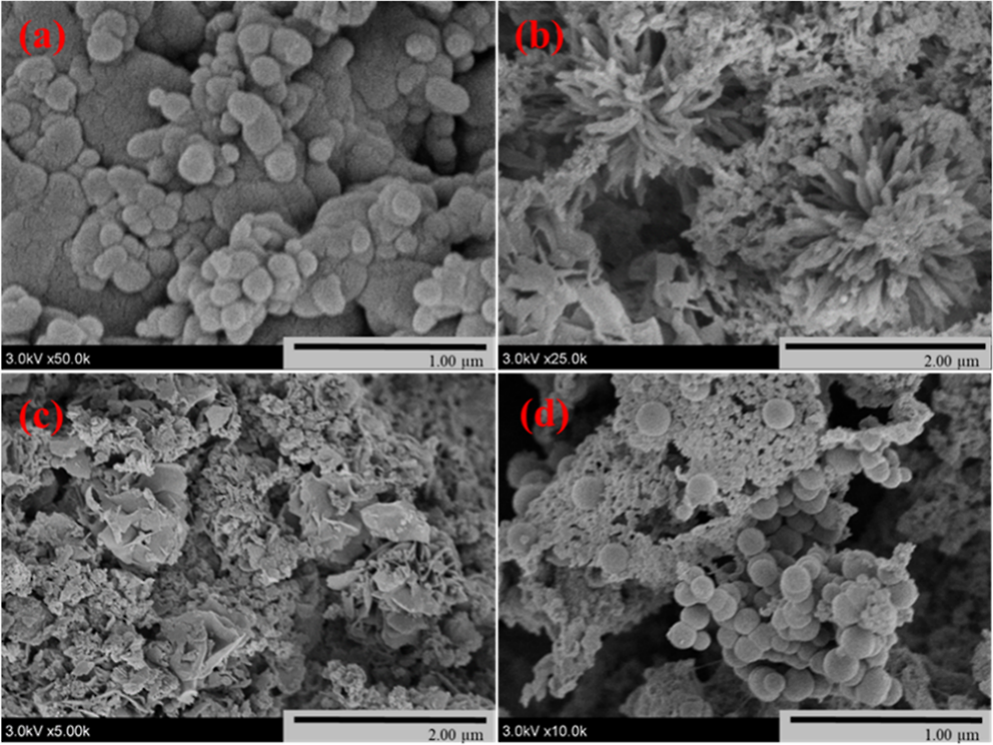
SEM micrographs of (a) HCOF, (b) TpPA, (c) TpBD, and (d) TpBDDA COFs.

**Figure 4:**
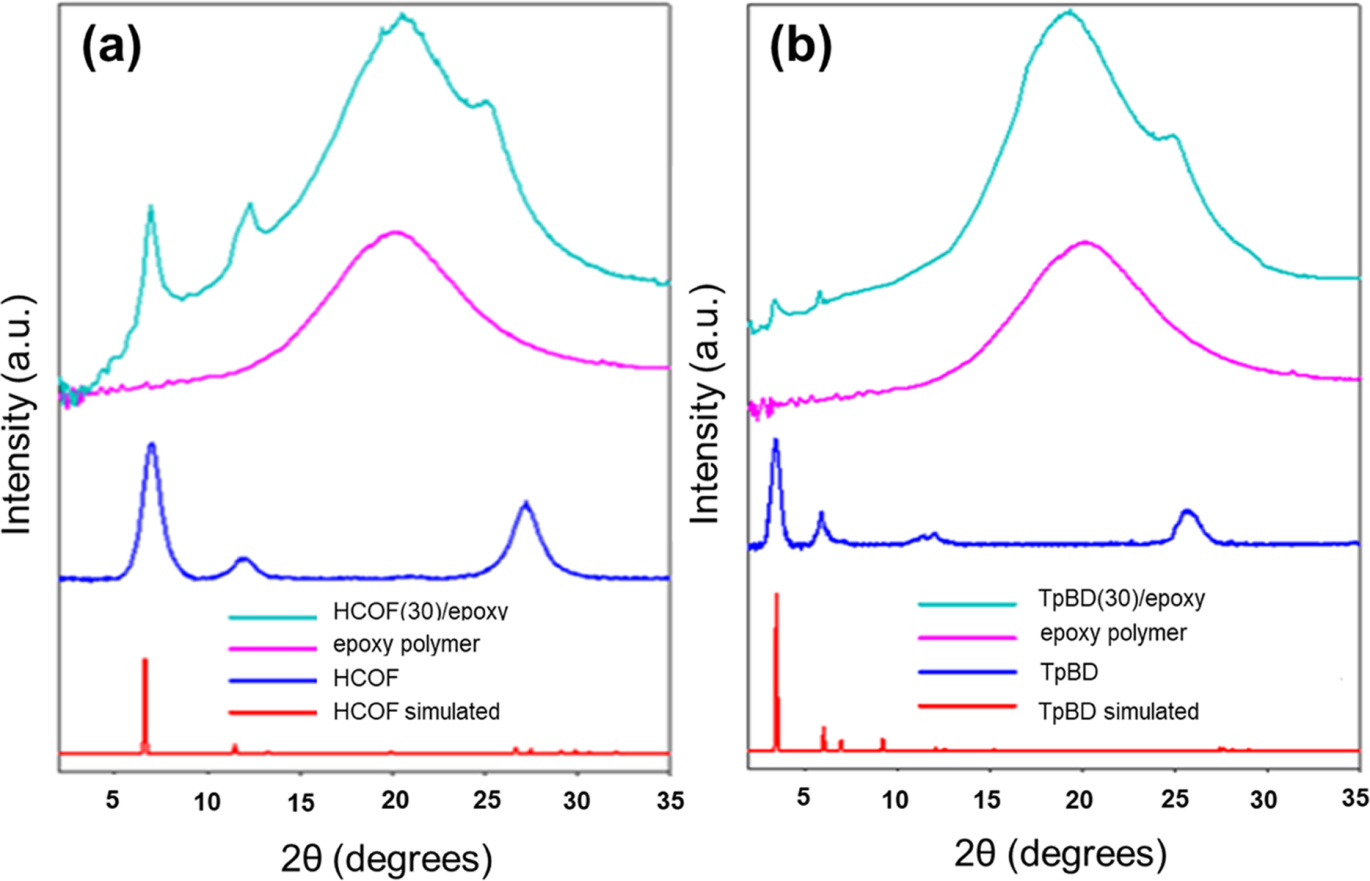
X-ray diffraction of (a) HCOF(30)/epoxy and (b) TpBD(30)/epoxy with the spectra of the COF, simulated spectra of the COF, and the spectra of the polyepoxy.

**Figure 5:**
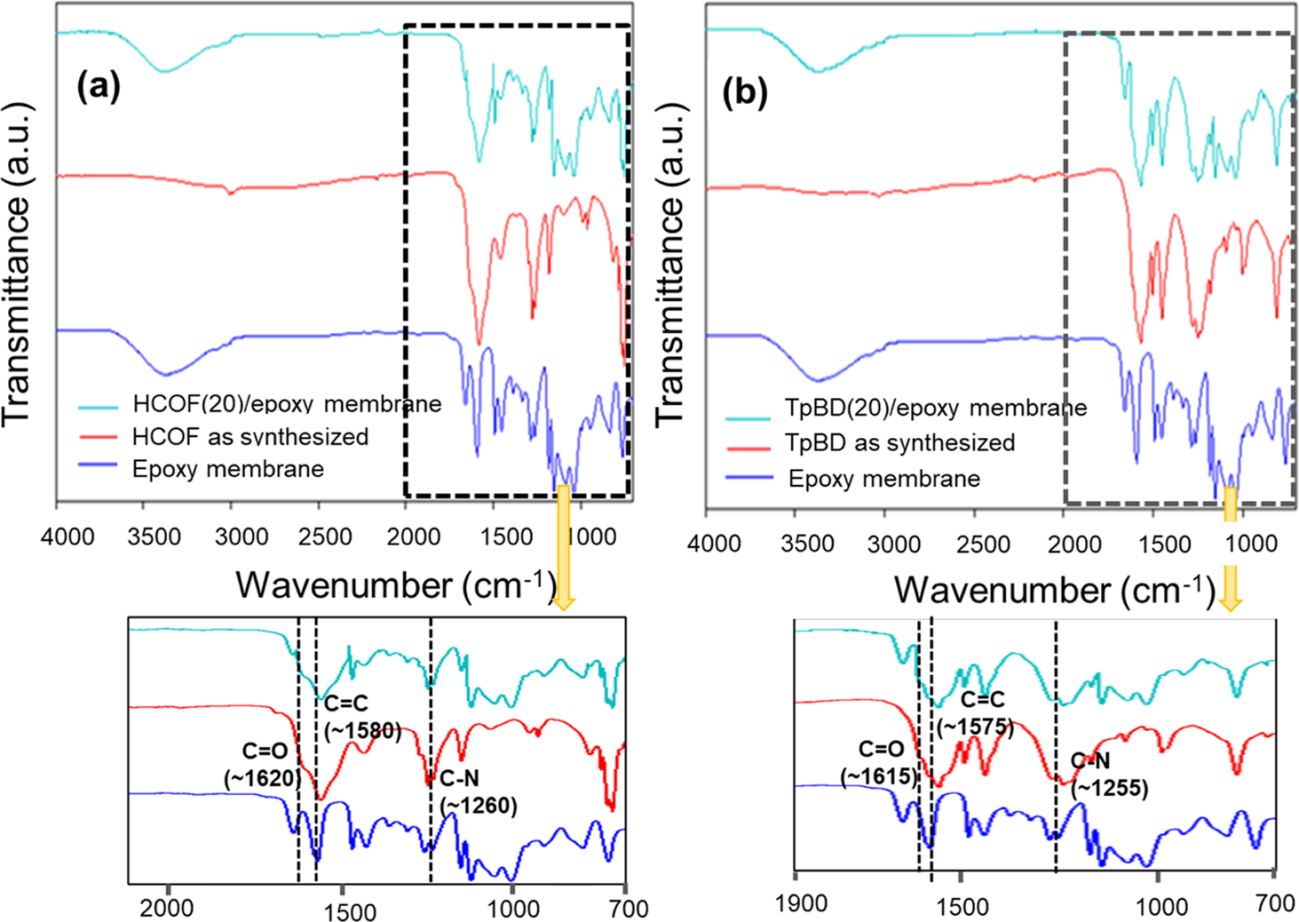
FT-IR spectra of (a) HCOF(20)/epoxy and (b) TpBD(20)/epoxy membranes with the spectra of the COF and polyepoxy.

The morphologies of the COFs were analyzed by SEM, PXRD, and IR spectroscopy and compared to prior work. SEM images (Figure 3) showed that the COFs crystallized with nanometer-sized dimensions. The energy dispersive spectrum of the TpPA COF showed that the COF contained a mix of C, N, and O (Figure S9). The COFs were crystalline, as shown by PXRD, and their spectra matched spectra from prior work and the corresponding simulated patterns (Figures 4 and S5). All of these spectra had a broad reflection at ∼25 degrees, which was assigned to the (001) plane and confirmed the synthesis of 2D COFs in a crystalline and π–π stacked form. The other peaks and their assignments for HCOF were located at 6.2° (100) and 11.5° (210).^54^ Similarly, for the TpBD COF, the most intense peak was visible at 3.3° (100) with other minor peaks at 6.3° (200) and 11.7° (210).^55^ Importantly, the PXRD spectra matched with the eclipsed (AA) stacking model, which indicated that the pores were aligned and formed a tunnel through the COFs.^55^ FT-IR spectra of the COFs showed bands associated with the C=O, C=C and C–N stretching modes, which was consistent with previously reported work (Figures 5 and S6).^51,53,54,56^

The surface areas and pore size distributions of the four COFs were found using Brunauer–Emmett–Teller analysis. The BET surface areas were 387, 498, 552, and 625 m^2^/g for the HCOF, TpPA, TpBD, and TpBBDA COFs, respectively, which agreed well with results reported in the literature (Figure S11). The pore size distributions of the COFs showed that they were well-ordered, with pore sizes of 1.41, 1.83, 2.25, and 3.42 nm for the HCOF, TpPA, TpBD, and TpBBDA COFs, respectively (Figure S12).

### Fabrication of COF/Polyepoxy Membranes

The COFs were incorporated into a polyepoxy matrix to synthesize COF(*n*)/epoxy mixed matrix membranes, where *n* was the percent loading by weight of the COFs in the polyepoxy. The polyepoxy was chosen based on prior work that showed that membranes fabricated from the monomers shown in Figure 1c had rapid flux for various chemicals.^19,26^ To synthesize the membranes, the resorcinol diglycidyl ether and the amine were first thoroughly mixed and placed under a slight vacuum to remove air bubbles. Next, either 10 or 20% by weight of a COF was added to the prepolymer and sonicated for 30 min to yield a suspension with the COFs well dispersed. Next, the COF/epoxy mixture was spin coated on a PZ membrane. The PZ membranes are a commercial poly(acrylonitrile) OSN membrane with a molecular weight cutoff of 30,000 g mol^–1^. The PZ membranes were used to provide structural support to the mixed matric membranes, and prior work demonstrated that they did not separate C18 FAs or FAMEs.^26^ Finally, the mixed matrix membranes were cured under a saturated atmosphere of DMF at 25 °C for 48 h. Eight mixed matrix membranes were fabricated using 10 or 20% COF, including HCOF(10)/epoxy, HCOF(20)/epoxy, TpPA(10)/epoxy, TpPA(20)/epoxy, TpBD(10)/epoxy, TpBD(20)/epoxy, Tp-BDDA(10)/epoxy, and TpBDDA(20)/epoxy. The top surface and cross-section of the TpPA(20)/epoxy membrane were characterized by SEM. The top surface of the membrane was flat and largely featureless; the cross-section of the membrane showed that the polyepoxy was in contact with the solid support and was approximately 90 μm thick (Figures S7,S8).

The COF(*n*)/epoxy membranes were characterized by wide angle X-ray diffraction (WXRD), as shown in Figures 4 and S5. Polyepoxy membranes without COFs were fabricated on the PZ solid support to investigate peaks due to these materials. Polyepoxy membranes in the absence of COFs showed a broad WAXD peak from 12–30°, which was consistent with their amorphous nature (Figure 4).^57^ The COF(*n*)/epoxy membranes were analyzed by WAXD to ensure the crystalline structure of HCOF, TpPA, TpBD, and TpBDDA COFs embedded inside the epoxy matrix. The WAXD patterns of the mixed matrix membranes all possessed a large, broad peak from 12–30° for the amorphous polyepoxy component and peaks that were present in the PXRD of the COFs before incorporation into the membrane. These results confirmed that the COFs were encapsulated within the polyepoxy matrix and that the COFs maintained their structures.^45^

The structural integrity of the COFs in the polyepoxy was also confirmed via FT-IR spectroscopy. In prior work, it was shown that the reaction of amines and epoxides to yield polyepoxy membranes was complete after 48 h^26^ This result was confirmed by examining the FT-IR spectrum of a polyepoxy membrane fabricated without COF. The epoxide peaks at approximately 860 and 910 cm^–1^ were completely missing after 48 h, which confirmed that the epoxides had reacted. In addition, the disappearance of the N–H peaks around 3375 and 3310 cm^–1^ and the appearance of a broad peak for the OH at 3500 cm^–1^ provided further evidence that reactions between the amines and epoxides were completed.^26^ In the mixed matrix membranes, the appearance of peaks at approximately 1550 cm^–1^ was due to the C=C bond of the reacted Tp, the peaks at 1480 and 1265 cm^–1^ were due to the C=C (Ar) and C–N functional groups.^51^ Further, the average thickness of the COF(*n*)/epoxy membranes was approximately 55 ± 10 μm.

### Separation of C18 FAMEs Using COF(*n*)/Epoxy Membranes

**Figure 6:**
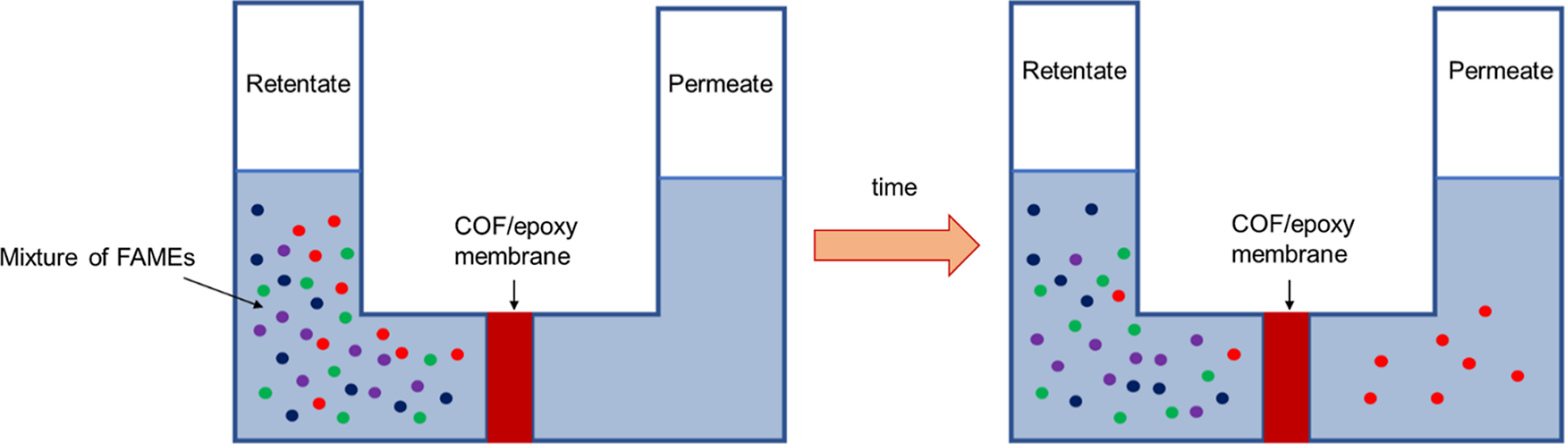
Membrane was placed between two glass arms. At the start of a separation, solvent was added to both sides of the membrane, with C18 FMAEs only added to the retentate side. Chemicals with a fast flux permeated the membrane rapidly, while chemicals with a slower flux permeated at a slower rate.

The separation of C18 FAMEs by osmosis was completed, as shown in Figure 6. A COF(*n*)/epoxy membrane was placed vertically between two solvent reservoirs. On the retentate side of the membrane, the FAMEs were added in a solvent, and another solvent was added to the permeate side of the membrane. Solvents on both sides of the membrane were well mixed throughout the separation using stir bars. In addition to the FAMEs, *p*-nitrobenzaldehyde was used as an internal standard in each experiment, and a small amount of BHT was added as an antioxidant to the retentate and permeate sides. At periodic times an aliquot was removed from the retentate and permeate, the solvent was evaporated, and the composition of the FAMEs was measured by NMR spectroscopy. The absolute flux of C18 FAMEs for each membrane was determined using a mathematical model as reported in the literature.^26^ The separation of the four most important vegetable oil FAMEs were investigated: methyl stearate (C18:0), methyl oleate (C18:1), methyl linoleate (C18:2), and methyl linolenate (C18:3).

The flux of the FAMEs was measured in triplicate for each of the eight mixed matrix membranes. In Table 1, the absolute flux for each FAME is shown, and for each membrane, the flux of methyl linolenate was set equal to one, and the relative flux for the other FAMEs were listed. Several observations emerged, including that for each membrane, the higher the degree of unsaturation, the slower the flux; methyl stearate had the most rapid flux and methyl linolenate had the slowest flux. The fluxes of the FAMEs were faster when COF(10)/epoxy membranes were used compared to COF(20)/epoxy membranes. The largest difference in flux between methyl stearate and methyl linolenate was for the TpPA(20)/epoxy membrane. This membrane had pore sizes of 1.8 nm, and the difference in flux was lower when the pore size of the COF was smaller or larger than this value.

**Table 1:** Absolute and Relative Fluxes of C18 FAMEs through Different COF(*n*)/Epoxy Membranes

	absolute flux {10^–7^} (mol/h cm^2^)				flux of chemicals relative to methyl linolenate			
COF(*n*)/epoxy membranes	methyl stearate	methyl oleate	methyl linoleate	methyl linolenate	methyl stearate	methyl oleate	methyl linoleate	methyl linolenate
HCOF(20)/epoxy	10.4 ± 1.18	5.7 ± 1.32	4.7 ± 0.67	2.2 ± 1.58	4.7	2.5	2.1	1
TpPA(20)/epoxy	20.1 ± 0.04	10.1 ± 0.28	8.7 ± 1.29	3.4 ± 0.68	5.9	2.9	2.6	1
TpBD(20)/epoxy	21.3 ± 1.12	11.4 ± 1.12	9.9 ± 0.67	4.8 ± 0.58	4.5	2.4	2.1	1
TpBDDA(20)/epoxy	22.7 ± 0.02	12.4 ± 0.06	11.2 ± 0.27	6.1 ± 0.03	3.8	2.0	1.8	1
HCOF(10)/epoxy	12.5 ± 0.34	8.2 ± 1.09	5.8 ± 0.85	3.9 ± 0.94	3.2	2.1	1.5	1
TpPA(10)/epoxy	23.5 ± 0.14	15.6 ± 1.09	10.9 ± 0.52	6.2 ± 0.44	3.8	2.5	1.8	1
TpBD(10)/epoxy	25.1 ± 0.23	19.7 ± 1.03	15.1 ± 1.01	9.3 ± 1.05	2.7	2.1	1.6	1
TpBDDA(10)/epoxy	28.2 ± 0.63	23.6 ± 0.77	19.2 ± 1.01	12.4 ± 1.01	2.3	1.9	1.5	1
epoxy membrane (without a COF)	32.6 ± 0.76	29.4 ± 0.87	26.8 ± 0.62	25.6 ± 0.89	1.2	1.2	1.1	1
S1(20)/epoxy	30.2 ± 0.43	28.4 ± 0.71	27.5 ± 0.47	24.7 ± 0.53	1.2	1.1	1.1	1

Two control experiments were completed to investigate the effect of the polyepoxy matrix and COFs in the separations. Polyepoxy membranes were fabricated without a COF, and the separation of the FAMEs was investigated (Table 1). The fluxes of the FAMEs were similar for this membrane, which provided further proof that the COFs were necessary for the separations. To investigate if noncovalent interactions between the FAMEs and the COFs were responsible for the difference in flux of the FAMEs, a fragment of a COF was synthesized and labeled S1 (Figure S10). This fragment was incorporated into a polyepoxy membrane, and the separation of the FAMEs was investigated (Table 1). The FAMEs had very similar flux through the S1(20)/epoxy membrane and were nearly identical to the small differences in flux found with membranes fabricated only from polyepoxy. This experiment demonstrated that the pores in the COFs, rather than the functional groups in the COFs, were responsible for the separation of FAMEs.

The ability to recycle the membranes was investigated using the TpPA(20)/epoxy membrane. The FAMEs were separated, and then the membrane was washed thoroughly in CH_2_Cl_2_. Next, a new set of FAMEs were separated, and then the membrane was washed in CH_2_Cl_2_ before a third separation of FAMEs was separated (Table S9). The flux and separation of the FAMEs decreased with each cycle.

### Separation of C18 FAs Using COF(*n*)/Epoxy Membranes

Mixtures of FAs were separated using three COF(20)/epoxy membranes using the same method, as described for the separation of the FAMEs except for the choice of solvents (Table 2). All the FAMEs were soluble in DCM, so this solvent was used for those separations, but stearic acid was not soluble in DCM. To separate the FAs, a 75/25% DCM/MeOH solvent mixture was used.

**Table 2:** Absolute and Relative Fluxes of C18 FAs through Different COF(*n*)/Epoxy Membranes

	absolute flux {10^–7^} (mol/h cm^2^)				flux of chemicals relative to linolenic acid			
COF(*n*)/epoxy membranes	stearic acid	oleic acid	linoleic acid	linolenic acid	stearic acid	oleic acid	linoleic acid	linolenic acid
HCOF(20)/epoxy	6.7 ± 1.23	3.7 ± 1.1	2.4 ± 1.21	1.8 ± 1.18	3.7	2.0	1.3	1
TpPA(20)/epoxy	13.9 ± 1.3	6.6 ± 1.01	4.3 ± 1.09	3.1 ± 0.69	4.5	2.2	1.4	1
TpBD(20)/epoxy	15.1 ± 1.02	11.3 ± 0.58	6.4 ± 0.52	5.2 ± 0.29	2.9	2.1	1.2	1
S1(20)/epoxy	15.6	13.7	12.2	12.0	1.3	1.2	1.0	1
epoxy membrane	16.2	15.0	14.8	12.3	1.3	1.2	1.2	1

The results in Table 2 showed that the general trends for the separation of FAs were similar to those obtained for the separation of FAMEs. For each membrane, the flux decreased as the degree of unsaturation increased, and the mixed matrix membrane fabricated with TpPA (pore size = 1.8 nm) had the largest difference in flux between linolenic acid and stearic acid. When the pore size was smaller or larger than 1.8 nm, the differences in flux decreased.

In control experiments, the separation of FAs through polyepoxy membranes and S1(20)/epoxy membranes showed little difference in flux (Table 2). These experiments provided further evidence that the pores of COFs were responsible for the separation of the FAs.

### Permeation of Methyl (*Z*)-5-Octenoate and Ethyl Octanoate through the TpPa(20)/Epoxy Membrane

The permeation of methyl (*Z*)-5-octenoate (C8:3) and ethyl octanoate (C8:0) were investigated to determine if differences in molecular weights affected the flux of FA esters (Figure S10). Methyl (*Z*)-5-octenoate has one cis-pi bond similar to methyl oleate, but its molecular weight is 156.2 g mol^–1^, and methyl oleate has a molecular weight of 310.5 g mol^–1^. The flux of methyl (*Z*)-5-octenoate was 2.5× faster than the flux of methyl oleate through a TpPA(20)/epoxy membrane (Table S1). Similarly, the flux of ethyl octanoate (molecular weight: 172.2 g mol^–1^) was 1.8× faster than the flux of methyl stearate (molecular weight: 298.5 g mol^–1^) [Table S2]. Both experiments showed that the flux of FA esters had a small dependence on molecular weights.

### Origin of the Difference in Flux for FAMEs and FAs

The difference in flux was consistent for all membranes and showed that as the degree of unsaturation increased, the flux was slower for both FAs and FAMEs. The rate of diffusion of chemicals within a membrane correlates with their flux, and these rates are highly dependent on the size of chemicals and interactions of the chemicals with the membrane.^19,26,58,59^ Larger chemicals tend to have slower flux than smaller chemicals, and chemicals that interact strongly with the membranes also have a slow flux.^26,59^ Furthermore, the pore sizes of the membranes must be similar in size to the chemicals for effective separations. The separation of FAMEs is challenging because they have numerous different conformations with similar energies, so their sizes and shapes are rapidly changing. Because of these fluxional sizes and shapes, four different membranes were investigated that had different pore sizes on the size scale of the FAMEs.

Experiments that investigated the flux of FAs and FAMEs through membranes fabricated with the COF fragment S1 showed that the difference in flux was not due to interactions between the FAMEs and FAs. The COF fragment S1 contained the functional groups of the COFs, but it had no effect on the separation of the FAMEs and FAs. The difference in flux was likely due to slight differences in the conformations and sizes of the chemicals. It is noteworthy that the FAMEs and FAs had a <2.2% difference in molecular weight and highly fluxional structures that made determining their sizes challenging.

In prior work by others, the radii of gyration, end-to-end distances, and characteristic ratios were investigated in silico for selected FAMEs (Table 3).^13^ The end-to-end distance was defined as the average of the separation between the two ends of the FAMEs, and the characteristic ratio was a measure of the flexibility of the FAMEs. The larger the value for the characteristic ratio, the longer the average extended chain was for a FAME. The unsaturated FAMEs possessed lower values for the characteristic ratios than the saturated FAMEs, which indicated that they possessed more compact structures as expected based on the cis-pi bonds that add curvature to FAMEs. It is important to note that TpPA/epoxy membranes had pore sizes of 1.8 nm for the COFs, which were similar to the end-to-end distances of the FAMEs reported in Table 3. Membranes fabricated from HCOF (pore size: 1.3 nm) had pore sizes that were smaller than the FAMEs, and those fabricated from TpBD (pore size: 2.3 nm) and TpBDDA (pore size: 3.4 nm) had pore sizes that were larger than the FAMEs and provided less ability to separate them. The TtPA COFs had pore sizes that were best optimized to separate the FAMEs.^13^

**Table 3:** Radii of Gyration, End to End Distances, and Characteristic Ratios for FAMEs at 333 K was Reported in the Literature^13^

type of FAMEs	radii of gyration (Å)	end to end distances (Å)	characteristic ratios (unitless)
methyl stearate (C18:0)	6.9 ± 0.6	20.8 ± 3.1	9.4 ± 0.3
methyl oleate (C18:1)	5.4 ± 0.6	14.9 ± 4.0	5.2 ± 0.5
methyl linoleate (C18:2)	5.4 ± 0.7	14.2 ± 3.8	4.8 ± 0.5
methyl linolenate (C18:3)	5.3 ± 0.5	13.4 ± 0.5	4.4 ± 0.5

An aspect that further complicates the analysis is that the FAMEs and FAs were dissolved in CH_2_Cl_2_ (for FAMEs) and 75/25% CH_2_Cl_2_/CH_3_OH (for FAs) at loadings of 2 grams of FAME or FAs per 60 mL of solvent. When the solvent was changed, the difference in flux for methyl stearate and methyl linolenate changed substantially for the TpPA(20)/epoxy, as shown in Table S4. Although the trend of faster flux for methyl stearate than methyl linolenate held for each solvent, there was a significant difference in the ratio for different solvents. The change in solvent affects how the FAMEs, FAs, and COFs are solvated and affects their structures, such as their radii of gyration.^60−63^ In addition, the change in solvent can affect whether FAMEs flux primarily through the polyepoxy (with little difference in flux for FAMEs) or the COFs. The difference in the ratio of flux for the different solvents in Table S4 did not correlate with the dielectric constant or dipole moments of the solvents. Due to these challenges, a full understanding of the molecular basis for the difference in flux could not be determined.

### Multiple Extractions to Purify a FAME from a Mixture of FAMEs

The ability of the membranes to separate and purify methyl linoleate was investigated using multiple separations (Table 4). An equimolar mixture of methyl stearate and methyl linoleate (5.35 g of each) was added to the retentate side of a membrane separation as before, and after a period of time, the solvent and FAMEs on the permeate side were removed and replaced with fresh solvent. The composition of the retentate had increasingly higher ratios of methyl linoleate to methyl stearate due to the slower flux of methyl linoleate. The purity of methyl linoleate was 50% in the initial mixture, 77% after the fourth cycle, and 85% after the fifth cycle. The permeate samples were combined, and they had a final composition of 33% methyl linoleate and 67% methyl linoleate. Over 98% of the FAMEs that were added were found in either the retentate or permeate, which demonstrated that only a small amount was found in the membrane or otherwise lost. These experiments demonstrated the potential of these membranes to separate and purify FAMEs.

**Table 4:** Composition of C18 FAMEs in the Retentate Side of the Membrane after Each Cycle

	amount of FAME in retentate					
chemical	initial amount (mmol)	cycle 1 (mmol)	cycle 2 (mmol)	cycle 3 (mmol)	cycle 4 (mmol)	cycle 5 (mmol)
methyl stearate	18.0	10.12	8.25	6.12	3.21	1.71
methyl linoleate	18.0	14.34	13.14	12.08	10.84	9.86
purity of methyl linoleate	50%	59%	61%	66%	77%	85%

## Conclusions

Industrial methods to inexpensively separate mixtures of C18 FAs and FAMEs are based on technology that is over four decades old, and new methods are needed to produce streams highly enriched in one FA or FAME. Membrane separations offer a solution to this challenge, but C18 FAMEs and FAs have very similar sizes and shapes, and all prior polymeric membranes were ineffective at separating them. In this article, we described how mixed matrix membranes composed of nanometer-sized COFs embedded within polyepoxy effectively separated C18 FAMEs and FAs based on their degrees of unsaturation. The key difference between these membranes and polymeric membranes was that polymeric membranes have broad distributions of pore sizes, but the crystalline structures of the COFs have narrow, nanometer-sized, and well-defined pores that provided the selectivity for separation. The COF with a pore size of 1.8 nm has the largest difference in flux, and COFs with smaller or larger pore sizes were less effective at separating the FAMEs and FAs. This work points to new directions for using OSN membranes composed of materials with crystalline pores, such as COFs or metal organic frameworks, to separate FAMEs, FAs, and other chemicals with slight differences in their molecular weights.

## Supporting Information Available

NMR spectra of Tp; PXRD diffraction patterns for TpPA and Tp-BDDA COFs; IR spectra of TpPA and Tp-BDDA COFs; absolute fluxes and the relative fluxes of methyl (*Z*)-5-octanoate through TpPA (20)/epoxy membranes; absolute fluxes and relative fluxes of ethyl octanoate through TpPA (20)/epoxy membranes; BET analysis of COFs; and SEM micrographs of the membranes (PDF)
