# Supplementary material for: Separation of C18 Fatty Acid Esters and Fatty Acids Derived from Vegetable Oils Using Nanometer-Sized Covalent Organic Frameworks Incorporated in Polyepoxy Membranes
Source: ACS Appl Nano Mater. 2023 Apr 10;6(8):6715–25. doi: 10.1021/acsanm.3c00442 (PMC10153466; doi:10.1021/acsanm.3c00442)
Supplement: Supplementary file 1 — an3c00442_si_001.pdf [file an3c00442_si_001.pdf]

Supporting Information

Separation of C18 Fatty Acid Esters and Fatty Acids Derived from Vegetable Oils Using Nanometer-Sized Covalent Organic Frameworks Incorporated in Polyepoxy Membranes

Nimesh P.R. Ranasinghe Arachchige, Nathan W. Xiong, Ned B. Bowden*

Department of Chemistry, University of Iowa, Iowa City, Iowa, 52242, United States of America

ned-bowden@uiowa.edu

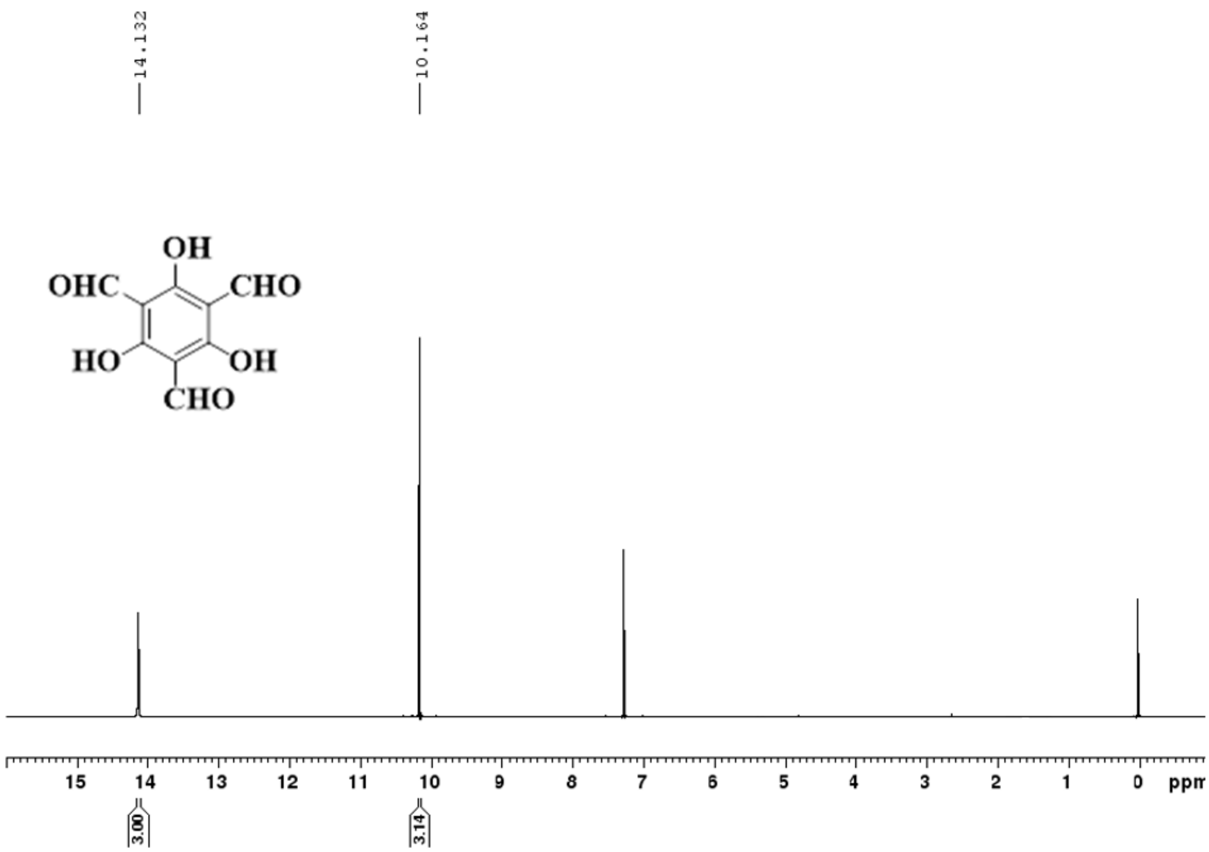

Figure S1. ^{1}H NMR spectrum of 3,5-triformylphloroglucinol (Tp)

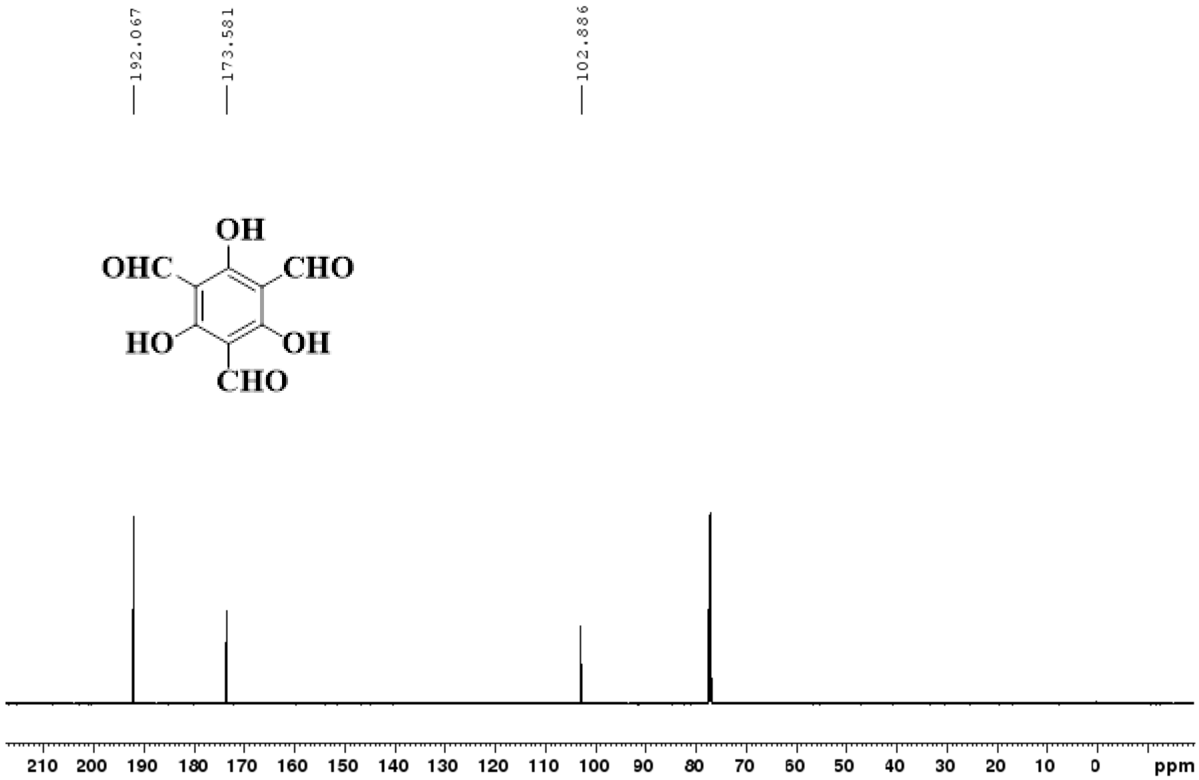

Figure S2. ^{13}C NMR spectrum of 3,5-triformylphloroglucinol (Tp)

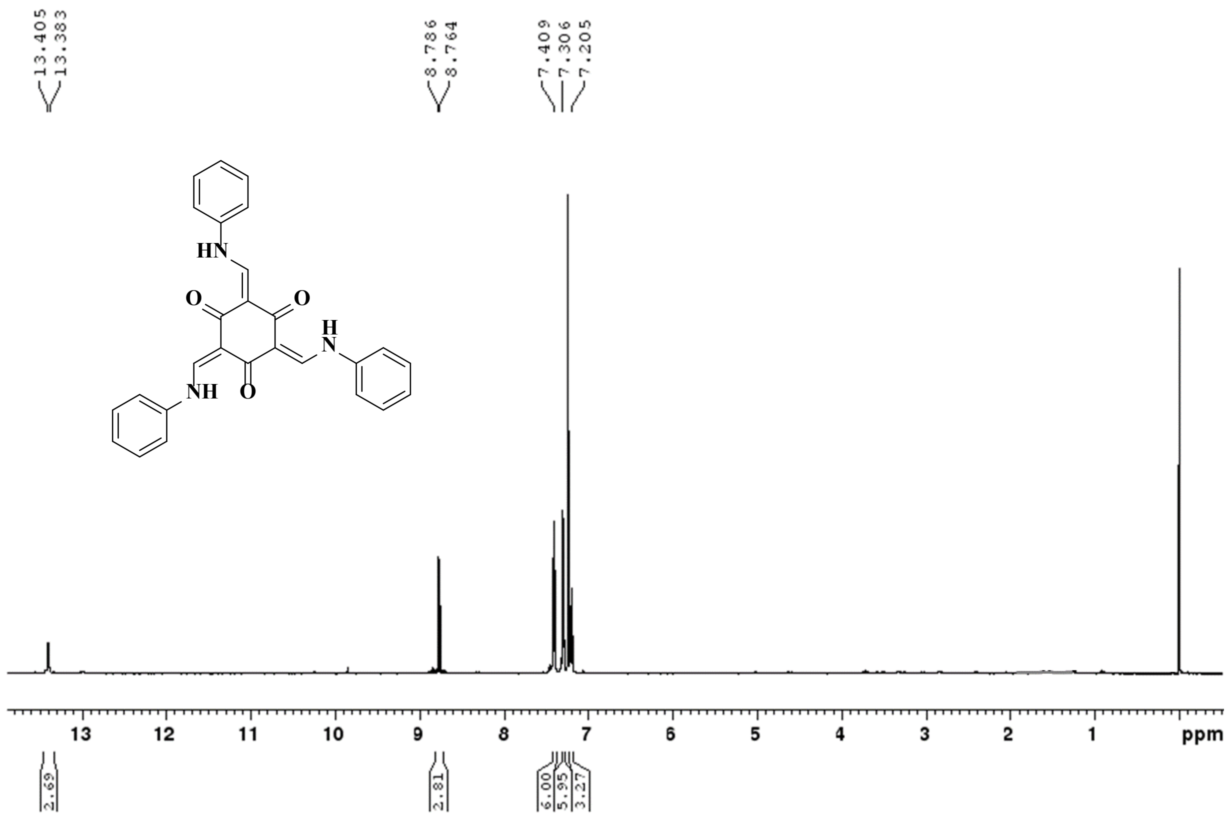

Figure S3. ^{1}H NMR spectrum of COF fragment (S1).

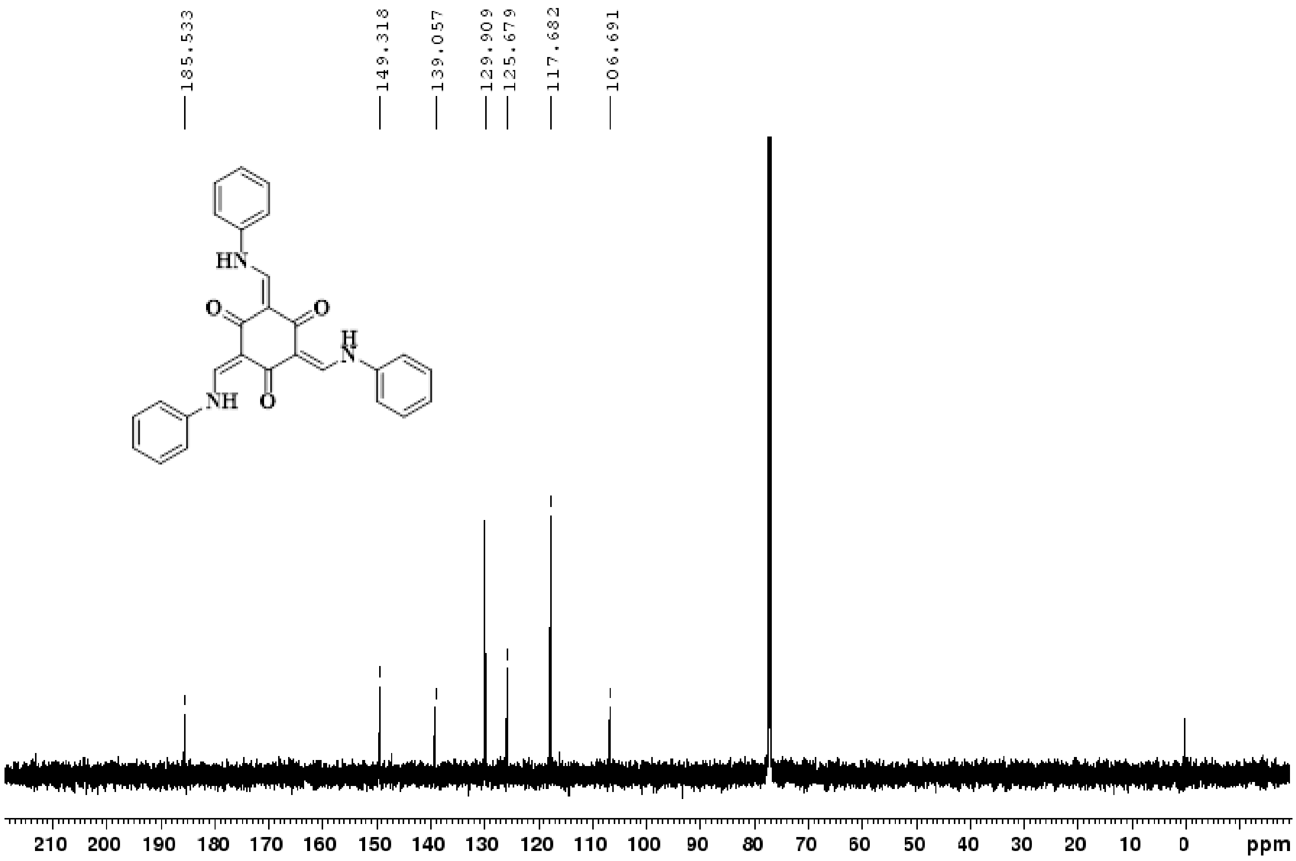

Figure S4. ^{13}C NMR spectrum of COF fragment (S1).

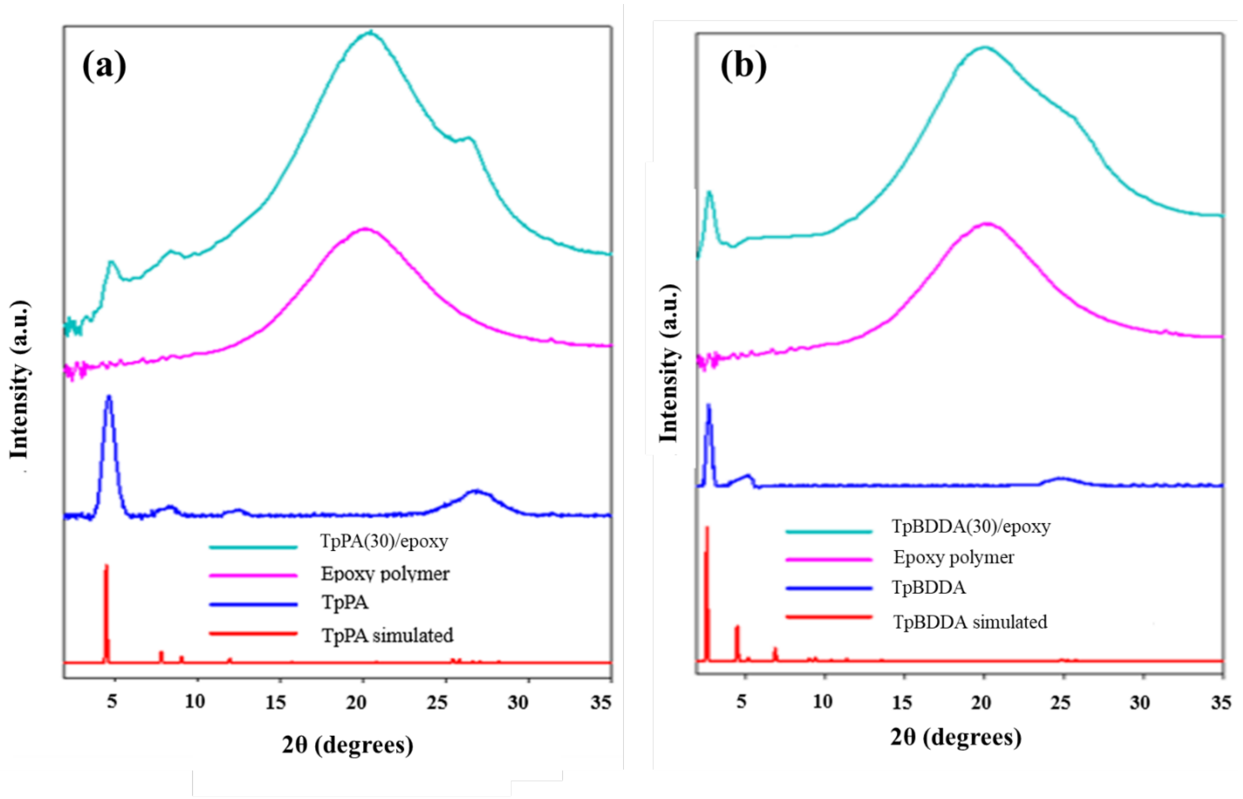

Figure S5. X-ray diffraction of (a) TpPA(30)/epoxy and (b) TpBDDA(30)/epoxy with the spectra of the COF, simulated spectra of the COF, and the spectra of the polyepoxy.

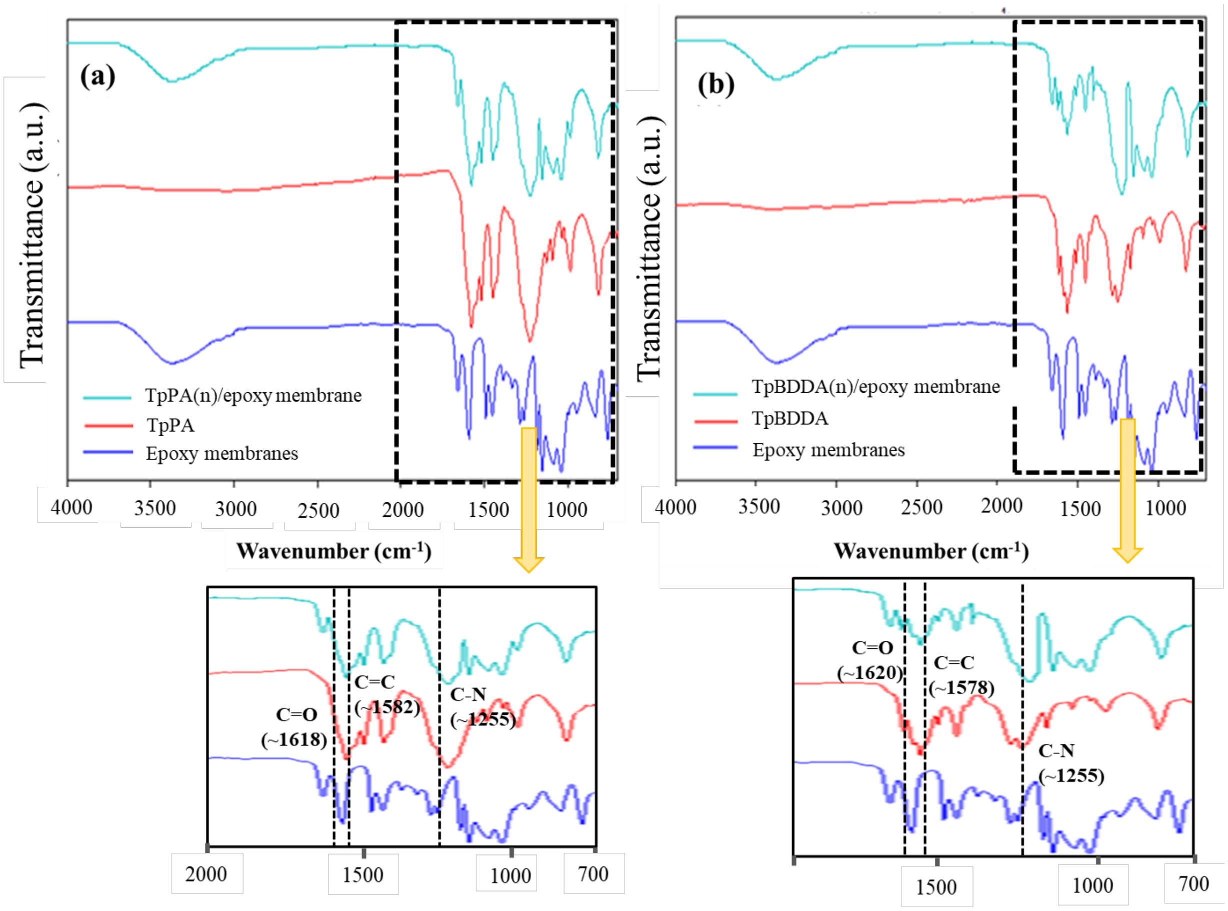

Figure S6. FT-IR spectra of (a) TpPA(20)/epoxy and (b) TpBDDA(20)/epoxy with the spectra of the COF, and the spectra of the polyepoxy.

Table S1. Absolute fluxes and the relative fluxes of methyl (*Z*)-5-octanoate through TpPA (20)/epoxy membranes in comparison to fluxes of methyl oleate is shown.

Membrane	Absolute flux {10^{-7}} (mol/h cm^2)		Relative flux of chemicals	
	**Methyl oleate**	**Methyl (*Z*)-5-octanoate**	**Methyl oleate**	**Methyl (*Z*)-5-octanoate**
TpPA (20)/epoxy	10.2	25.5	1	2.5

Table S2. Absolute fluxes and the relative fluxes of ethyl octanoate through TpPA (20)/epoxy membranes in comparison to fluxes of methyl stearate is shown.

Membrane	Absolute flux {10^{-7}} (mol/h cm^2)		Relative flux of chemicals	
	**Ethyl octanoate**	**Methyl stearate**	**Ethyl octanoate**	**Methyl stearate**
TpPA (20)/epoxy	36.4	20.1	1.8	1

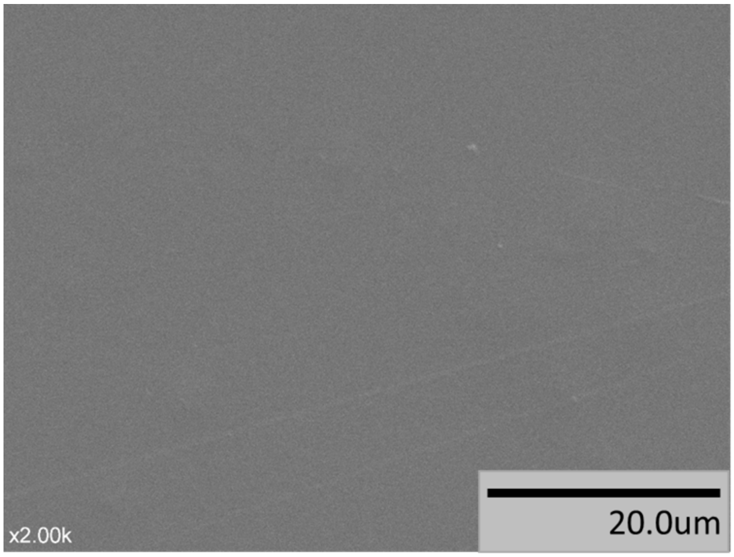

Figure S7. A scanning electron micrograph of the top surface of the TpPA(20)/epoxy membrane is shown.

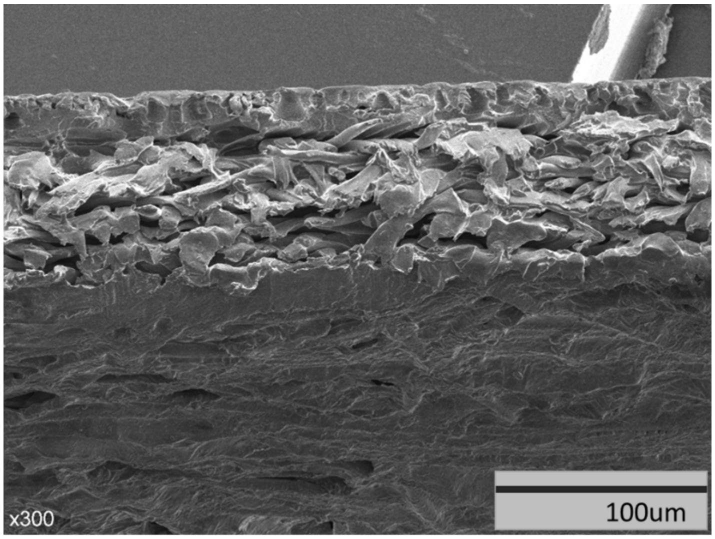

Figure S8. A scanning electron micrograph of the cross-section of an TpPA(20)/epoxy membrane cast on a solid support is shown

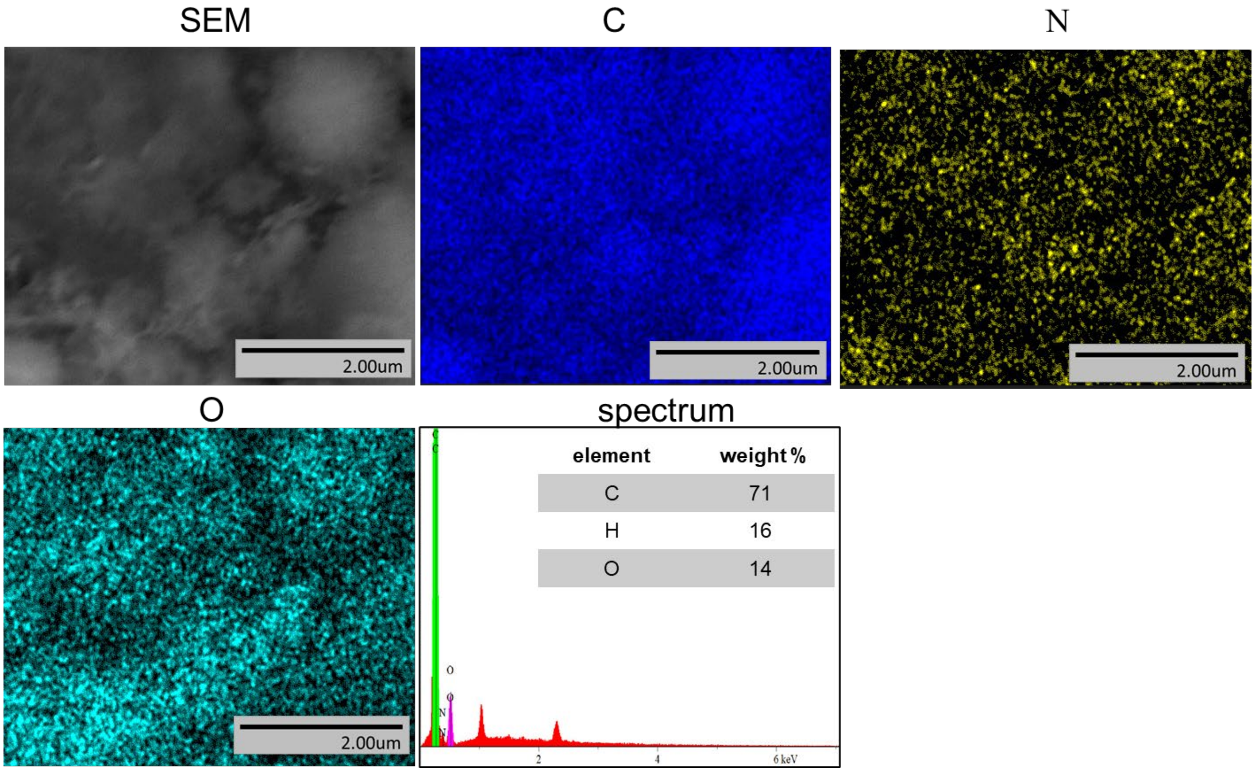

Figure S9. The EDS elemental mapping of the TpPA COF is shown.

Table S3. The flux of FAMEs through the TpPA(20)/epoxy membrane is shown. Between each cycle the membrane was rinsed with CH_2Cl_2.

Cycle number	Absolute flux $\{10^{-7}\}$ (mol/h cm^2)				Flux of FAMEs relative to methyl linolenate			
	MS	Omega-9	Omega-6	Omega-3	MS	Omega-9	Omega-6	Omega-3
1	20.1	10.1	8.7	3.4	5.9	2.9	2.6	1
2	14.1	5.3	3.8	2.8	5.0	1.9	1.4	1
3	8.2	3.8	2.5	2.1	3.8	1.7	1.1	1

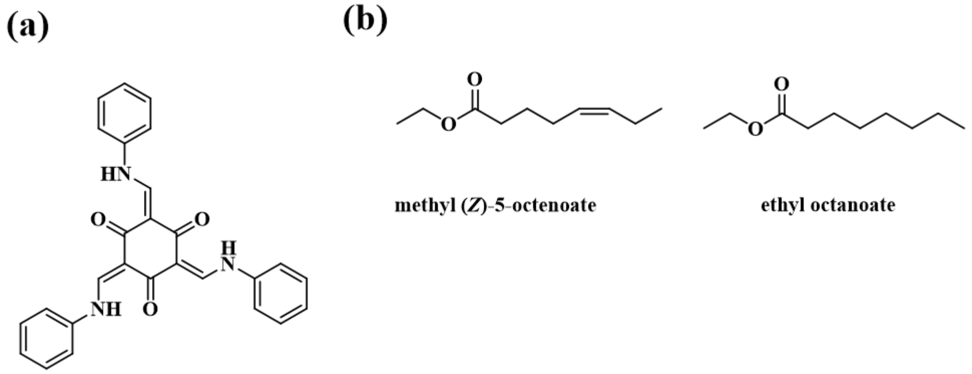

Figure S10. a) The structure of the small fragment (S1) is shown. b) The structures of methyl (*Z*)-5-octenoate and ethyl octanoate are shown.

Table S4. The relative flux of methyl stearate relative to methyl linolenate with different solvents.

Item	Solvent			
	CH_2Cl_2	$CHCl_3$	CH_3OH	Acetone
Ratio for flux of methyl stearate and methyl linolenate	5.9	3.8	2.7	1.8
Dielectric constant	8.93	4.81	32.7	20.7
Dipole moment	1.14	1.15	2.87	2.69

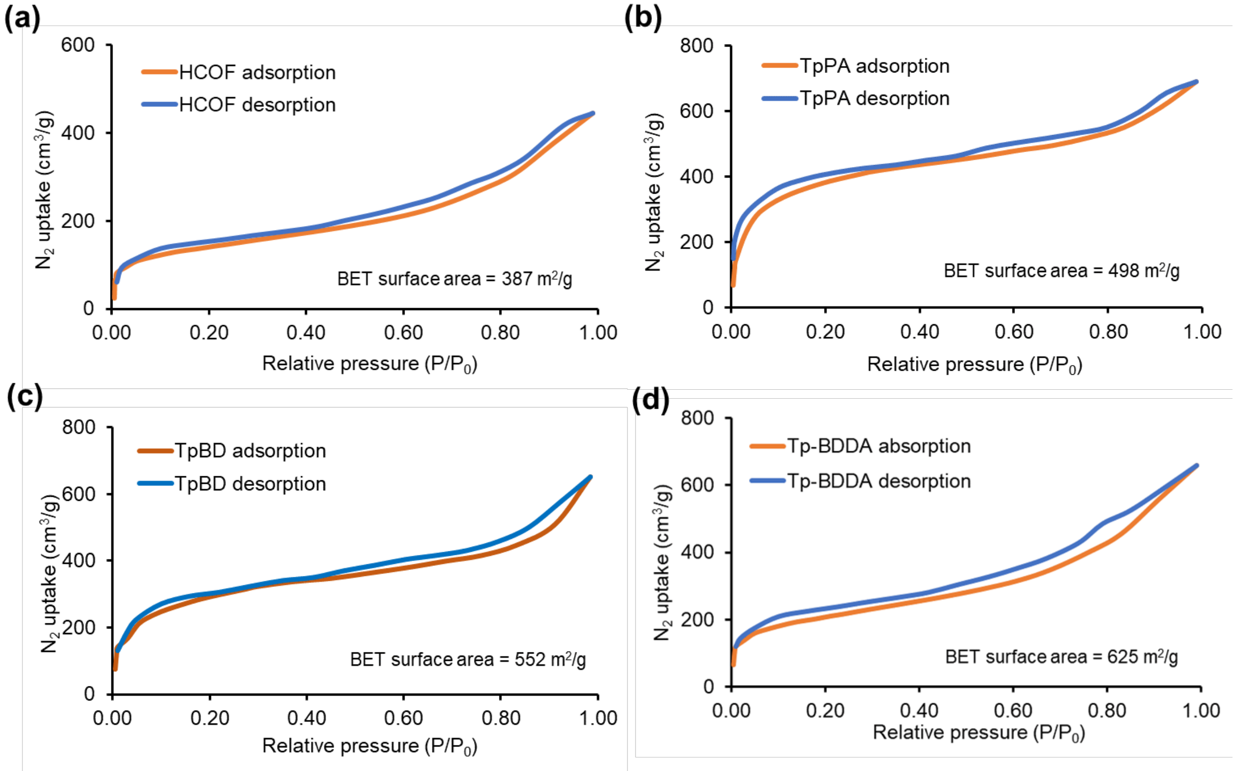

Figure S11. The BET analysis of the COFs were completed and the surface areas were completed.

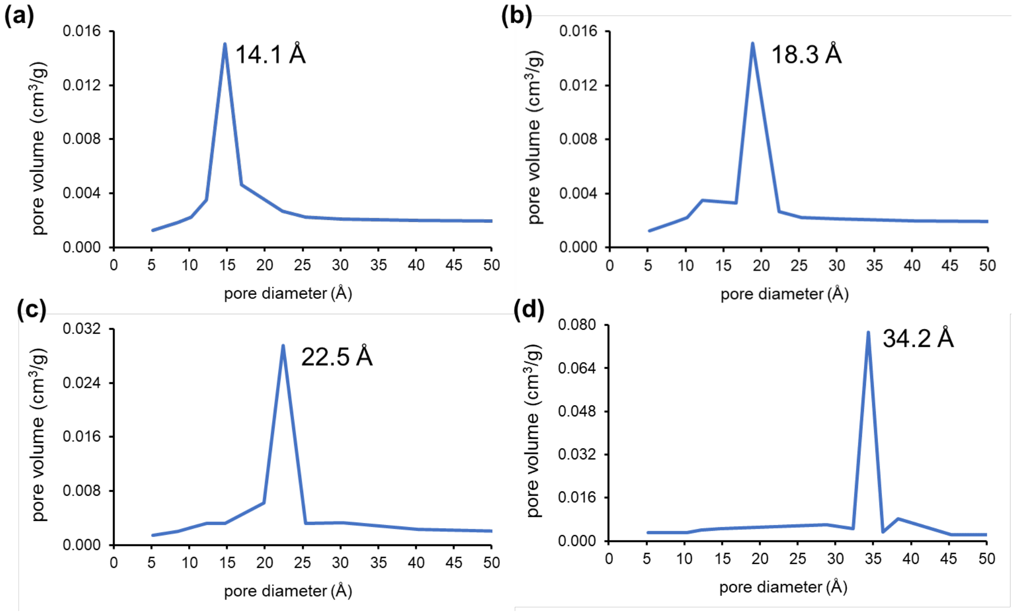

Figure S12. The pore sizes of the a) HCOF, b) TpPA, c) TpBD, and d) TpBBDA COFs were obtained from BET analysis. The numbers represent the pore diameter for the top of each peak.
